# Naturally acquired visceral leishmaniosis in a captive white-naped mangabey (*Cercocebus lunulatus)* in Spain

**DOI:** 10.1007/s11259-025-10894-7

**Published:** 2025-09-19

**Authors:** Sergio Villanueva-Saz, María Eugenia Lebrero, Jacobo Giner, Salvador Marín Lillo, Rafael Guerra, Xavier Roca-Geronès, Roser Fisa, Alicia de Diego, José Luis Arnal Bernal, Pablo Quílez, Álex Gómez, Diana Marteles

**Affiliations:** 1 Departamento de Patología Animal, Universidad de Zaragoza, C. de Miguel Servet, 177, Zaragoza, 50013 Spain; 2 Instituto Agroalimentario de Aragón-IA2 (Universidad de Zaragoza-CITA), Zaragoza, Spain; 3 Clinical Immunology Laboratory, Universidad de Zaragoza, Zaragoza, Spain; 4 AAP Primadomus, Alicante, Spain; 5 Wales Ape & Monkey Sanctuary, Caehopkin, Abercrave, UK; 6 Centro De Conservación Zoo Córdoba, Córdoba, Spain; 7 Departamento de Biología, Sanidad y Medioambiente, Universitat de Barcelona, Barcelona, Spain; 8 Centro de Investigación Biomédica de Aragón (CIBA), Instituto Aragonés de Ciencias de La Salud (IACS), Zaragoza, Spain; 9 Exopol. Pol. río Gállego D14, San Mateo de Gállego, Zaragoza, 50840 Spain

**Keywords:** *Leishmania infantum*, *Cercocebus lunulatus*, Captive, Spain

## Abstract

*Leishmania infantum* infection has recently been identified in non-human primates in Spain, including clinically affected individuals from critically endangered species such as the Bornean orangutan (*Pongo pygmaeus pygmaeus*). Despite these emerging cases, clinicopathological data on leishmaniosis in captive wildlife remain limited. Here, we report the first confirmed case of clinical visceral leishmaniosis in a zoo-captive white-naped mangabey (*Cercocebus lunulatus*) in Spain. The affected animal presented with progressive weight loss, generalized lymphadenopathy, polyclonal gammopathy, and cytological evidence of pyogranulomatous lymphadenitis containing intralesional protozoal amastigotes consistent with *Leishmania* spp. The animal eventually died, and a detailed necropsy was performed. Moreover, to confirm *L. infantum* infection, histopathological analysis, serological assays, including immunofluorescent antibody test (IFAT), Western blot (WB), and enzyme-linked immunosorbent assay (ELISA), as well as polymerase chain reaction (PCR) and immunohistochemical analysis of affected tissues were conducted. Gross pathological examination revealed disseminated visceral involvement, characterized by multiple white nodules in the lungs, spleen, liver, and lymph nodes. Microscopically, these lesions corresponded to areas of coagulative necrosis surrounded by macrophage-rich inflammatory infiltrates, many of which contained protozoal amastigotes compatible with *Leishmania* spp. *L. infantum* infection was confirmed by all aforementioned serological tests, PCR, and immunohistochemistry. This case highlights the expanding host range of *L. infantum* and underscores the need to include leishmaniosis in the differential diagnosis of primates in zoological institutions, especially in endemic regions.

**Supplementary Information:**

The online version contains supplementary material available at 10.1007/s11259-025-10894-7.

## Background

Leishmaniosis is a zoonotic vector-borne disease caused by the protozoan *Leishmania infantum.* In veterinary science, the majority of published literature has focused on domestic animals, with the dog (*Canis lupus familiaris*) identified as the primary peridomestic reservoir host (Baneth et al. 2008). However, other domestic species, such as cats (*Felis catus*), may also serve as potential reservoirs of infection (Pennisi and Persichetti 2018). More recently, in Europe, *L. infantum* infection has been documented in mustelids, more specifically in domestic ferrets *(Mustela putorius furo*) (Villanueva-Saz et al. 2021), as well as in wild species such as the Eurasian otter (*Lutra lutra*) (Cantos-Barreda et al. 2020) and the European mink (*Mustela lutreola*) (Del Carmen Aranda et al. 2025).

This parasite is capable of naturally infecting a wide range of both domestic (Cardoso et al. 2021) and wild mammalian species (Ortega-García et al. 2019). However, the epidemiological role of many wildlife species as reservoirs remains unknown. Limited information is available regarding the presence of *L. infantum* infection in zoological and wildlife park environments. In Spain, infections have been noted in a number of captive species, namely in Bennett’s wallaby (*Macropus rufogriseus rufogriseus*) (Ramírez et al. 2013; Montoya et al. 2016), orangutan (*Pongo pygmaeus pygmaeus*) (Miró et al. 2018) and meerkat (*Suricata suricatta*) (Moraleda-Berral et al. 2025). In other European countries, such as Italy, *L. infantum* infection has also been reported in tigers (*Panthera tigris*) (Cavalera et al. 2020).

Under experimental conditions, infection with *Leishmania* spp. has been demonstrated in various non-human primates, both Sykes’ monkeys (*Cercopithecus mitis albogularis*) and baboons, resulting in low-grade infections lasting between four to eight months, followed be spontaneous recovery (Githure et al. 1986; Binhazim et al. 1993; Gicheru et al. 1995). Furthermore, a model of visceral leishmaniasis that closely resembles the human form of the disease, has been successfully reproduced in macaques (Porrozzi et al. 2006). These non-human infected primates developed a systemic infection characterized by symptomatology typical of human visceral leishmaniasis, including fever, diarrhea, weight loss, anemia, hypergammaglobulinemia, transient lymphocytosis, and enlarged lymph nodes, liver, and spleen (Porrozzi et al. 2006).

Although uncommon, natural infection and disease caused by *Leishmania* spp. has been reported in non-human primates. The confirmation of infection in both captive and wild populations was attained by means of serology, molecular testing, histopathology and immunohistochemistry (Malta et al. 2010; Lima et al. 2012; Rovirosa-Hernández et al. 2013; Souza et al. 2014; Bueno et al. 2017; Tinoco et al. 2018).

This report presents the first documented case of a naturally occurring disseminated visceral presentation of *L. infantum* infection in a captive white-naped mangabey (*Cercocebus lunulatus*), a species that is currently facing near extinction in Africa.

## Case presentation

### Case history

An 18-years-old captive White-naped mangabey (*Cercocebus lunulatus*) born in the Parc Zoologic of Barcelona and transferred to the Córdoba Zoo Conservation Center (Córdoba, Spain) at the age of 7-years-old was clinically examined because of progressive weight loss, general weakness and apathy with no other apparent clinical signs. This condition altered social dynamics in the group as he lost his social status. Neither the monkey´s medical history nor his annual health check ups revealed any potential signs of illness or disease. Furthermore, three months earlier the primate was anesthetized to suture a wound caused by a bite from a group member, and during the clinical examination, no symptomatology indicative of leishmaniosis was detected. However, the aggression suffered by the individual could be related to a change in the hierarchy within the existing group of males (three adults and on juvenile) which may have been related to a subclinical presentation of the disease, unfortunately bloodwork was not deemed necessary at the time of the intervention. Approximately one month before the animal´s death. On clinical examination the patient presented severe muscular atrophy, pale mucous membranes and there was evidence of an axillary lymph node enlargement (Supplementary material S1). The animal was anesthetized with dexmedetomidine (0.04 mg/kg) (Dexdormitor^®^ 0.5 mg/ml, Lab. Ecuphar) and ketamine (10 mg/kg) (Ketamidor^®^ 100 mg/ml, Lab. Karizoo), administered via a remote dart using a blowpipe. Under anesthesia, a fine needle aspirate from a lymph node was obtained and stained with Diff-Quick stain for cytological examination. Blood samples were obtained to perform serum protein electrophoresis analysis and serological tests. During anesthesia, the animal died due to an acute respiratory failure caused by a spontaneous pneumothorax, which was identified through postmortem radiography and confirmed by subsequent necropsy.

### Serum protein electrophoresis

Serum protein electrophoresis was performed by agarose gel electrophoresis (AGE) (Hydragel Kit 1–2, Sebia, Issy-les-Moulineaux, France). Serum was electrophoresed for 21 min at 92 V and stained with diluted amidoschwarz dye at pH 2 (4 g/L amidoschwarz dye and 6.7% ethylene glycol). The AGE procedure was conducted according to the manufacturer’s instructions and commercial human serum was used as a control (normal control serum; Sebia, Evry, France). The electrophoretic curve for each sample was displayed and read with a GELSCAN TM densitometry system (Sebia, Issy-les-Moulineaux, France). The electrophoretic curve for each sample was assessed using Phoresis software. Total protein concentration was measured by the automatic analyzer Catalyst One Chemistry Analyzer (Idexx, USA). Protein fractions were determined as a percentage of optical absorbance, and the absolute concentration g/dL was automatically calculated from the total serum protein concentration using a spectrophotometer. Albumin-to-globulin (A: G) ratios were also calculated. The same operator analyzed all samples (Marteles et al. 2025). Given the absence of information of a serum protein profile for the White-naped mangabey species, a serum sample collected from healthy White-naped mangabey was also included.

The affected animal exhibited an acute-phase response, characterized by an increase in α2-globulins and a polyclonal gammopathy, reflected by elevated γ- and β-globulin levels forming a broad base and wide peak, in comparison to the negative control of White-naped mangabey (*Cercocebus lunulatus*) which displayed a non-altered serum protein profile (Supplementary material S2).

### Serological analysis

For serological testing, three different in-house anti-*Leishmania infantum* antibody detection techniques were employed, namely indirect immunofluorescence antibody test (IFAT), enzyme-linked immunosorbent assay (ELISA) and Western Blot (WB) analysis. The indirect immunofluorescence antibody test (IFAT) for detecting anti-*Leishmania*-specific immunoglobulin G (IgG) antibodies was carried out using an anti-monkey IgG fluorescein-labeled conjugate, as previously described (Deniau et al. 2003), with a cut-off value of ≥ 1:100 to determine seropositivity. An ELISA for the detection of antibodies against *L. infantum* using a whole *L. infantum* promastigote antigen (MHOM/FR/78/LEM75 zymodeme MON-1) was performed on the diluted serum sample (1:100) following the protocol described previously (Alcover et al. 2021) including Protein A/G conjugated to horseradish peroxidase diluted 1:10,000. This conjugate interacts with immunoglobulin G in different mammal species, allowing the use of positive and negative controls from different species in the absence of controls for the species being serologically tested. Each plate included a panel of positive serum samples with a known antibody status (dog, cat, wild felids). By contrast, a serum sample from a healthy, non-infected, seronegative White-naped mangabey (*Cercocebus lunulatus*) from the same zoo, previously tested by EDTA-blood PCR and quantitative ELISA serology, was used as a negative control.The sample, positive control, calibrator and negative control were run in duplicate, with the cuff-off set at 0.200 optical density (OD) units. Finally, for WB analysis, anti-*Leishmania* antibodies were detected using a whole *L. infantum* promastigote antigen (MHOM/FR/78/LEM75 zymodeme MON-1), as described previously (Alcover et al. 2021). A serum sample was considered WB-positive if immunoreactivity against the 14 kDa and/or 16 kDa low-molecular-weight polypeptide fractions of the *L. infantum* antigen was observed. Additionally, the same serum from a healthy, non-infected, seronegative White-naped mangabey (*Cercocebus lunulatus*) was included as a negative control. Moreover, detection of the antibodies against Simian Immunodeficiency Virus (SIV) and Simian Retrovirus (SRV) by ELISA was performed in a private laboratory (Echevarne Laboratorios, Spain).

Anti-*Leishmania* antibodies were detected using IFAT, ELISA, and WB. In the case of IFAT, an antibody titer of 1:800 was observed. For the ELISA technique, an optical density (OD) value of 1.211 was detected for this clinical case. WB analysis demonstrated immunoreactivity against *Leishmania* antigen fractions of 14, 16, 18, 20, 24, 28, 30, 34, 36, 44, 46, 48, 50, 53, 55, 58, and 71 kDa (Supplementary Fig. 2). Moreover, the animal tested seronegative by ELISA for both SIV and SRV viruses.

### Pathological study

Cytological examination revealed a high cellularity comprised of macrophages, multinucleate giant cells and rare degenerate neutrophils in the background of an amorphous eosinophilic material with clear vacuoles. Some of the macrophages contained oval organisms within their cytoplasm. These organisms were characterized by an eccentric nucleus and a small amount of pale cytoplasm, measured approximately 3 to 4 am in diameter, compatible with *Leishmania* spp. (Supplementary material S1).

During necropsy lung, lymph nodes, spleen, liver and kidneys were sampled and then fixed in 10% neutral-buffered formalin, embedded in liquid paraffin, and 4 μm-thick sections were stained with hematoxylin and eosin (HE) and three other histochemical stains, Gram, Periodic Acid-Schiff (PAS) and Ziehl-Neelsen (ZN). To determine the presence of *Leishmania infantum* amastigotes in tissue sections, immunohistochemistry was performed, as previously described (Giner et al. 2020).

**Fig. 1:**
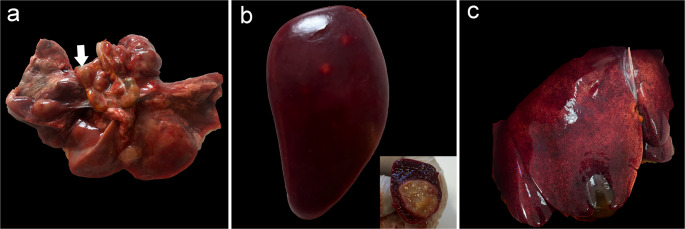
Gross lesions in a captive White-naped mangabey with visceral leishmaniosis caused by *Leishmania. infantum*. (**a**) Marked lymphadenomegaly in the mediastinal lymph nodes (arrow) and multiple whitish nodules within the pulmonary parenchyma. (**b**) Whitish nodules affecting the spleen. Inset: Sectioned nodules exhibited a yellowish and gelatinous appearance. (**c**) Liver showing mottled appearance (nutmeg liver) suggestive of chronic passive congestion

On gross post-mortem examination, the lungs and their mediastinal lymph nodes, axillary, preescapular and inguinal lymph nodes, spleen, and liver exhibited similar lesions, characterized by multifocal whitish nodules with internal granules, consistent with necrotic foci (Fig. 1).

**Fig. 2:**
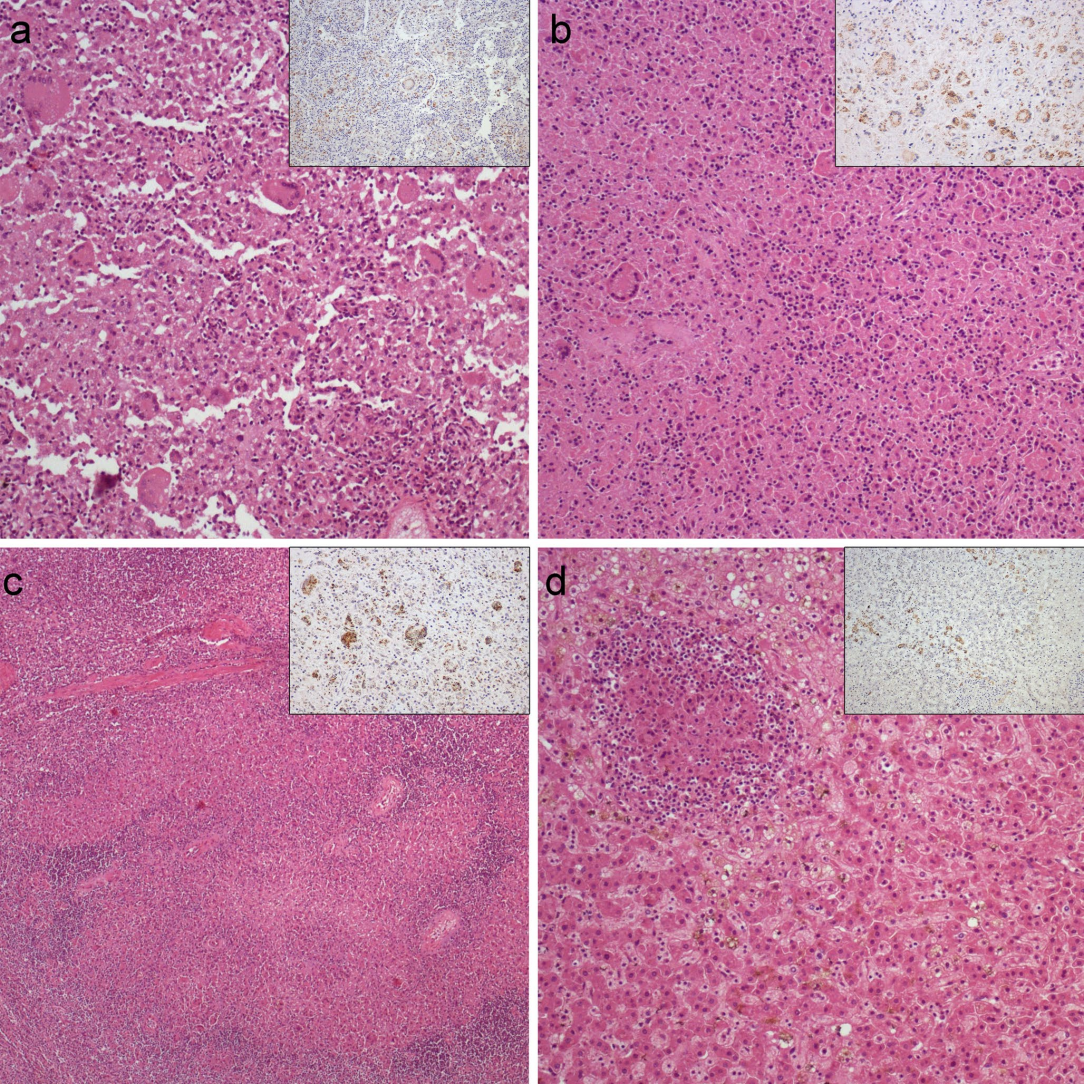
Microscopic lesions in a captive White-naped mangabey with visceral leishmaniosis caused by *Leishmania infantum*. Hematoxylin-eosin (HE). (**a**) Pyogranulomatous pneumonia with intralveolar giant cells. X20. (**b**) Mediastinal lymph node showing necrotazing and pyogranulomatous lymphadenitis with numerous giant cells. X10. (**c**) Spleen revealed multifocal granulomatous lymphadenitis. X10. (**d**) Liver exhibited foci of piogranulomatous inflammation in addition to diffuse sinusoidal ectasia and cholestasis. X20. Insets: Immunohistochemistry against *L. infantum* revealed positively stained amastigotes within macrophages and giant cells across all sampled tissues. X20

Histological examination revealed that the white nodules in the lungs, mediastinal lymph nodes, spleen, and liver corresponded to nodular foci of coagulative necrosis, surrounded and containing an inflammatory infiltrate composed primarily of macrophages, some of them multinucleated (foreign body and Touton types), along with viable and degenerated neutrophils, and fewer lymphocytes and plasma cells (Fig. 2). Some macrophages contained *Leishmania* spp. amastigotes within their cytoplasm. Gram, PAS and ZN were negative in all sampled tissues, ruling out visible bacteria, fungi or other parasites. However, *L. infantum* amastigotes within the macrophages cytoplasm stained positively (brown) by immunohistochemistry in lungs, mediastinal lymph nodes, spleen, and liver (Fig. 2).

### Molecular analysis

During necropsy lung, lymph node, spleen, and kidney tissue samples were analyzed using quantitative polymerase chain reaction (qPCR). For *L. infantum* analysis, DNA was extracted from 50 mg of tissue obtained from each organ using the High Pure PCR Template Preparation Kit (Roche Applied Science, Mannheim, Germany) according to the manufacturer’s instructions. The quantification of *Leishmania* kDNA was carried out by amplification of kinetoplast minicircle DNA sequences utilizing qPCR (Giner et al. 2020). Each amplification was performed in triplicate in 10 µl of reaction mixture containing 1× iTaq Supermix with ROX (Bio-Rad, Hercules, CA, USA), 15 pmol of direct primer Leim1 (5′-CTT TTC TGG TCC TCC GGG TAG G-3′), 15 pmol of reverse primer Leim2 (5′- CCA CCC GGC CCT ATT TTA CAC CAA-3′), 50 pmol of the labeled TaqMan probe Leim3 (5′- FAM-TTT TCG CAG AAC GCC CCT ACC CGC TAMRA-3′), and 2.5 µl of sample DNA. PCR was performed on an ABI Prism 7900 HT thermocycler (Applied Biosystems, Waltham, MA, USA) with 40 cycles at 94 °C and 55 °C, using fluorescence detection with a FAM dye. A non-template control was used in each run as the qPCR negative control. A 10-fold dilution series of DNA from promastigotes (MHOM/ES/04/BCN-61, *L. infantum*) was used for calibration, allowing the plotting of a standard curve. The PCR-RFLP methodology was carried out for the specific identification of *Leishmania* spp. The ITS1 region was amplified using LITSR and L5.8 S primers with a hybridization temperature of 53 °C. DNA amplification products were digested with restriction endonucleases HaeIII (New England Biolabs) at 37 °C, in accordance with the manufacturer’s recommendations. The digested products were subjected to electrophoresis in 2% agarose gel and visualized by a UV light Illuminator 2000 (BIO-Rad) (Schönian et al. 2003). Additionally, MatMet approach was performed, which involved partial sequencing of the mitochondrial cytB gene using the MinION nanopore platform. The objective of this methodology was to identify the *Leishmania* species present in various organs that had previously tested positive by species-specific qPCR, through partial sequencing of the kinetoplast cytB gene. Nucleic acids extracted from the tissues that tested positive were used for PCR amplification of a variable region of the cytB gene, which enables species-level discrimination. The primers leisinf_F* (5′-TGTGGTGTDTGTTTAGCRTGG-3′) and leisinf_R* (5′-CACAAAACCCATCTGAACTCA-3′), designed to detect multiple *Leishmania* species, were used under an annealing temperature of 58 °C. Library preparation was performed using the Native Barcoding Kit 24 V14 (SQK-NBD114.24), following the manufacturer’s instructions. Sequencing was conducted on the MinION platform (Oxford Nanopore Technologies, ONT, Oxford, UK) for up to 24 h, and demultiplexing of barcoded reads was carried out using MinKNOW software (v.24.02.8).

Finally, the detection of DNA from *Toxoplasma gondii*, pathogenic *Leptospira* spp., and *Salmonella enterica* was also performed for each organ. Firstly, tissue specimens were pre-treated using the MagMAX™ CORE Mechanical Lysis Module (Applied Biosystems, Austin, TX, USA), with two cycles at 6000 rpm for 30 s each in a MagNA Lyser (Roche Diagnostics, Penzberg, Germany). Nucleic acids were extracted and purified using the MagMAX™ CORE Nucleic Acid Purification Kit (Applied Biosystems, Austin, TX, USA), following the manufacturer’s instructions, with the KingFisher™ Flex instrument (Thermo Scientific, Rockford, IL, USA). The elution volume was adjusted from the user manual protocol to obtain 200 µL of elution buffer, ensuring sufficient volume for all required qPCR reactions. Once DNA was purified, several commercial qPCR assays from the EXOone series (Exopol, Zaragoza, Spain) were conducted. These kits provide quantitative detection of the mentioned pathogens via the 6-carboxyfluorescein (6-FAM) channel. To ensure quality control of the molecular detection process, all assays were also tested for amplification of an endogenous control using the HEX channel (hexachlorofluorescein). All qPCR reactions were performed using the QuantStudio™ 5 Real-Time PCR System (Applied Biosystems, Austin, TX, USA), according to the manufacturer’s protocols for each kit. Data were analyzed with QuantStudio Software v1.5.2, and results with a cycle threshold (Ct) ≤ 38 were considered positive. Additionally, affected tissues were screened for *Mycobacterium tuberculosis* complex DNA by PCR in a private laboratory (Echevarne Laboratorios, Spain).

**Fig. 3:**
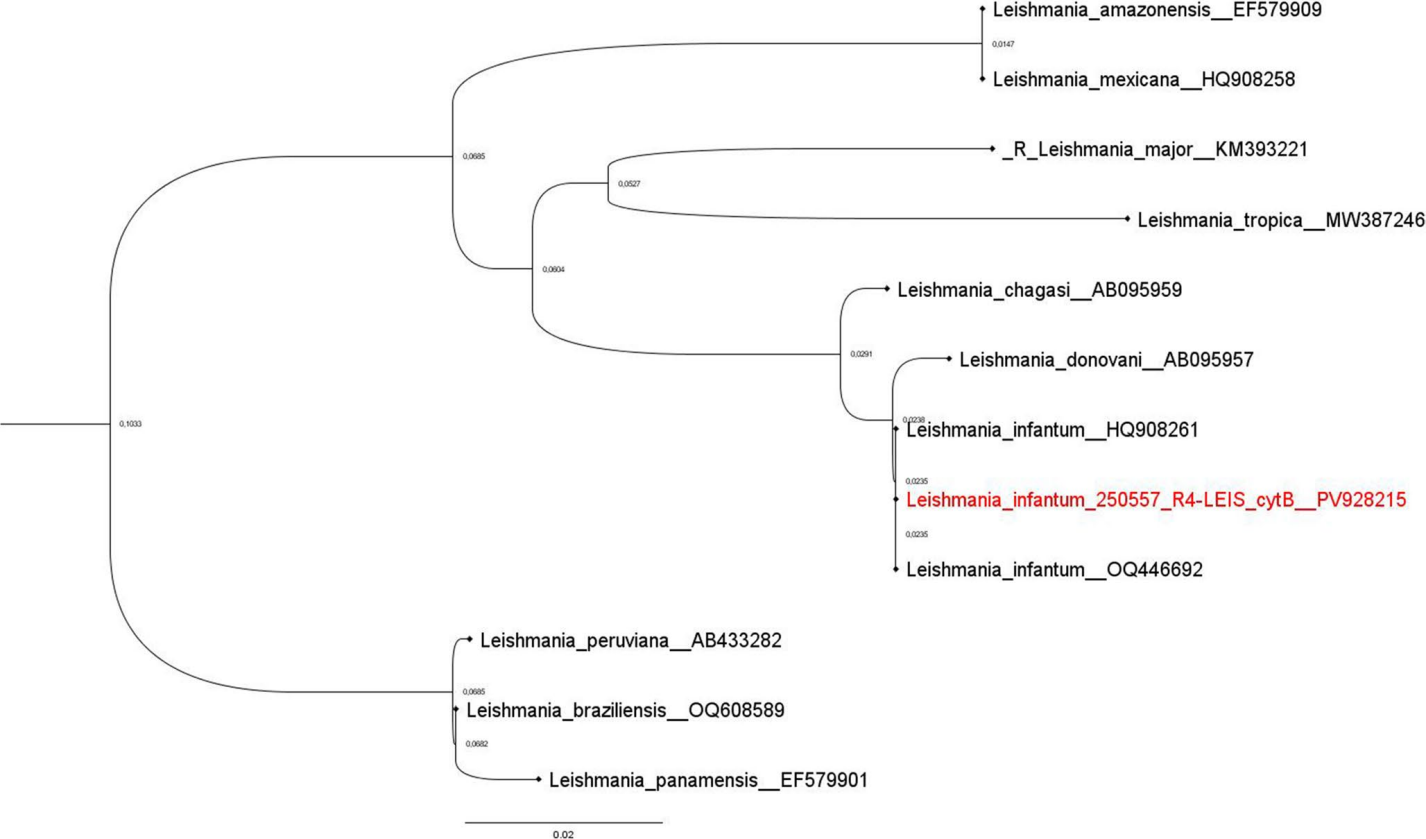
The phylogenetic tree was constructed using partial sequences of the mitochondrial cytochrome b (cytB) gene from various *Leishmania* species, with each sequence annotated by its respective GenBank accession number as indicated in the figure. The sequence obtained in this study, labeled as *Leishmania*_*infantum*_250557_R4-LEIS_cytB (GenBank accession number: PV928215), is highlighted in red to facilitate its identification. Multiple sequence alignment was performed using MAFFT software version 7, and phylogenetic relationships were inferred using the Neighbor-Joining (NJ) method with 1000 bootstrap replicates. The numerical values displayed near the nodes of the tree represent estimated genetic distances between lineages, reflecting the degree of nucleotide divergence within the aligned region of the cytB gene. The resulting topology clearly positions the sequence within the *Leishmania infantum* clade, thereby confirming its molecular identification as *L. infantum* and supporting its genetic affiliation with other representative strains of this taxon

*Leishmania* DNA was detected in three of the four analyzed organs (lymph node, spleen and lung). The higher concentration of parasites was observed in the spleen (1.4 × 10^4^ parasites/g of tissue), while the lymph node and lung showed lower concentrations (1.1 × 10^2^ and 1.7 × 10^3^ parasites/g, respectively). After the PCR-RFLP analysis, *L. infantum* was identified in all positive tissues by the visualization of species-specific patterns. The cytB gene sequences obtained from all positively tested organs were 100% identical to one another, indicating consistent detection of the same *Leishmania* genotype across tissues. Furthermore, these sequences exhibited 100% identity with multiple *Leishmania infantum* cytB gene entries in the NCBI GenBank database, confirming species identification. The resulting consensus sequence has been deposited in GenBank under the accession number PV928215 (Fig. 3). No *T. gondii*, *Leptospira* spp., *Salmonella* spp. and *Mycobacterium tuberculosis* complex DNA was detected in the four analyzed organs.

## Discussion and conclusions

This study represents the first clinicopathological description of visceral leishmaniosis in a captive White-naped mangabey, with involvement of the lungs, mediastinal lymph nodes, spleen, and liver. *Leishmania infantum* infection was confirmed through serology, immunohistochemistry, and qPCR analysis. However, the origin of the *L. infantum* infection was not identified. Futhermore, the most common immunosuppressive viruses were discarded by ELISA.

In this case, *L. infantum* caused pyogranulomatous pneumonia, lymphadenitis, splenitis, and hepatitis. These results are consistent with previous studies in *Leishmania* spp. infected non-human primates, where lymph nodes, liver, kidney, lung, small intestine and bone marrow were affected by the parasite (Malta et al. 2010; Montoya et al. 2016; Miró et al. 2018; Barbero-Moyano et al. 2024). Based on organ-specific parasite quantification by PCR, the highest concentration of parasites was observed in the spleen (1.4 × 10⁴ parasites/g of tissue), followed by the lung (1.7 × 10³ parasites/g), and lymph nodes (1.1 × 10² parasites/g). These results were corroborated by immunohistochemistry. In this case, a notable tropism of *L. infantum* for the lung was observed, compared to a lower parasite load in the analyzed lymph nodes. When compared with the known pathogenesis of leishmaniosis in domesticated dogs, cats, and ferrets (Cardoso et al. 2021), this clinical case can be classified as a disseminated visceral form of the disease.

Other studies have observed cutaneous and ocular affection by *L. infantum* (Moraleda-Berral et al. 2025; Montoya et al. 2016). This parasite is known to display a higher tissue tropism in other animal species, such as dogs, particularly in endemic regions (Menezes-Souza et al. 2014). Both wild and captive non-human primates, as well as humans, can become infected with this parasite under immunosuppressive conditions, as previously reported (Gicheru et al. 1995). In this case, the animal was seronegative to SIV and SRV. However, the advanced age of the animal and the possibility of the presence of other non-tested pathogens in this case study could have resulted in an immunosuppressive state of the animal. Moreover, the stress induced by the mentioned bite from a group member could influence the clinical progression of leishmaniosis. Therefore, this case highlights the need for monitoring and control of *L. infantum* and *Phlebotomus perniciosus* in Spanish zoos housing captive Bennett’s wallabies, orangutans, meerkats and White-naped mangabeys in order to reduce the risk of zoonotic transmission (Ramírez et al. 2013; Montoya et al. 2016; Miró et al. 2018; Moraleda-Berral et al. 2025).

In this case, the affected animal exhibited a polyclonal gammopathy, a finding previously described in multiple animal species. Co-infection with another immunosuppressive virus could have influenced the protein electrophoresis profile; however, recent studies have shown that animals co-infected with two or more pathogens do not exhibit significant variations in their proteinograms (Marteles et al. 2025). Although in this study hematologic and biochemical analysis was not performed. Anemia, neutropenia or pancytopenia, hypoalbuminemia and elevated transaminases have been observed in *Leishmania*-affected non-human primates (Miró et al. 2018).

Serologic diagnosis is important, especially in symptomatic animals, as in this case, where antibody titers are high (Gicheru et al. 1995; Malta et al. 2010). In instances of generalized lymphadenopathy, fine-needle aspiration for cytological examination and PCR-based analyses should be considered key diagnostic approaches. In fulminant cases of *L. infantum* infection, a thorough necropsy is essential, with collection of target tissues such as spleen, lymph nodes, liver, and lungs for histopathological, immunohistochemical, and PCR-based evaluation. The spleen is considered one of the most important organs in visceral leishmaniosis in primates (Ramírez et al. 2013; Miró et al. 2018; Moraleda-Berral et al. 2025). In infected non-human primates, screening for immunosuppressive viruses such as retroviruses, hepatitis viruses, or herpesviruses should be performed using serological methods.

In conclusion, this study provides the first description of the clinicopathological findings of *L. infantum* infection in a captive White-naped mangabey, without evidence of immunosuppression. This case reinforces the importance of implementing *L. infantum* surveillance and control strategies in captive non-human primates to prevent potential zoonotic transmission.

## Supplementary Information

Below is the link to the electronic supplementary material.

Supplementary Material 1 (DOCX 1.86 MB)

## Data Availability

No datasets were generated or analysed during the current study.

## References

[CR1] Alcover MM, Basurco A, Fernandez A et al (2021) A cross-sectional study of *Leishmania infantum* infection in stray cats in the City of Zaragoza (Spain) using serology and PCR. Parasit Vectors 14:178. 10.1186/s13071-021-04682-w 33766113 10.1186/s13071-021-04682-w PMC7992781

[CR2] Baneth G, Koutinas AF, Solano-Gallego L, Bourdeau P, Ferrer L (2008) Canine leishmaniosis - new concepts and insights on an expanding zoonosis: part one. Trends Parasitol 24:324–330. 10.1016/j.pt.2008.04.001 18514028 10.1016/j.pt.2008.04.001

[CR3] Barbero-Moyano J, Martínez R, Gonzálvez M et al (2024) Monitoring of *Leishmania infantum* in captive non-human primates in Spain. Res Vet Sci 180:105425. 10.1016/j.rvsc.2024.105425 39342921 10.1016/j.rvsc.2024.105425

[CR4] Binhazim AA, Shin SS, Chapman WL, Olobo J (1993) Comparative susceptibility of African green monkeys (*Cercopithecus aethiops*) to experimental infection with *Leishmania leishmania donovani* and *Leishmania leishmania infantum*. Lab Sci 43:37–47 8459677

[CR5] Bueno MG, Catão-Dias JL, de Oliveira Laroque P et al (2017) Infectious diseases in Free-Ranging blonde capuchins, Sapajus flavius, in Brazil. Int J Primatol 38:1017–1031. 10.1007/s10764-017-9994-5

[CR6] Cantos-Barreda A, Navarro R, Pardo-Marín L et al (2020) Clinical leishmaniosis in a captive Eurasian otter (*Lutra lutra*) in spain: a case report. BMC Vet Res 16:312. 10.1186/s12917-020-02509-x 32854701 10.1186/s12917-020-02509-x PMC7450804

[CR7] Cardoso L, Schallig H, Persichetti MF, Pennisi MG (2021) New epidemiological aspects of animal leishmaniosis in europe: the role of vertebrate hosts other than dogs. Pathogens 10:307. 10.3390/pathogens10030307 33800782 10.3390/pathogens10030307 PMC8000700

[CR8] Cavalera MA, Iatta R, Laricchiuta P et al (2020) Clinical, haematological and biochemical findings in Tigers infected by *Leishmania infantum*. BMC Vet Res 16:214. 10.1186/s12917-020-02419-y 32571332 10.1186/s12917-020-02419-y PMC7310479

[CR9] Del Carmen Aranda M, Villora J, Giner J et al (2025) A longitudinal study on the detection of anti-*Leishmania* antibodies in a captive European Mink (*Mustela lutreola*) population and their correlation with serum protein electrophoresis. Res Vet Sci 185:105541. 10.1016/j.rvsc.2025.105541 39837108 10.1016/j.rvsc.2025.105541

[CR10] Deniau M, Cañavate C, Faraut-Gambarelli F, Marty P (2003) The biological diagnosis of leishmaniasis in HIV-infected patients. Ann Trop Med Parasitol 97:115–133. 10.1179/000349803225002598 14678639 10.1179/000349803225002598

[CR11] Gicheru MM, Olobo JO, Kariuki TM, Adhiambo C (1995) Visceral leishmaniasis in Vervet monkeys: immunological responses during asymptomatic infections. Scand J Immulo 41:202–208. 10.1111/j.1365-3083.1995.tb03554.x 10.1111/j.1365-3083.1995.tb03554.x 7863267

[CR12] Giner J, Basurco A, Alcover MM, Riera C et al (2020) First report on natural infection with *Leishmania infantum* in a domestic ferret (*Mustela putorius furo*) in Spain. Vet Parasitol Reg Stud Rep 19:100369. 10.1016/j.vprsr.2020.100369 10.1016/j.vprsr.2020.100369 PMC7103921 32057396

[CR13] Githure JI, Shatry AM, Tarara R, Chulay JD, Suleman MA, Chunge CN, Else JG (1986) The suitability of East African primates as animal models of visceral leishmaniasis. Trans R Soc Trop Med Hyg 80:575–576. 10.1016/0035-9203(86)90146-x 3810791 10.1016/0035-9203(86)90146-x

[CR14] Lima VM, Santiago ME, Sanches L, daC, Lima BD (2012) Molecular diagnosis of *Leishmania amazonensis* in a captive spider monkey in bauru, São paulo, Brazil. J Zoo Wildl Med 43:943–945. 10.1638/2012-0059R1.1 23272368 10.1638/2012-0059R1.1

[CR15] Malta MCC, Tinoco HP, Xavier MN, Vieira ALS, Costa ÉA, Santos RL (2010) Naturally acquired visceral leishmaniasis in non-human primates in Brazil. Vet Parasitol 169:193–197. 10.1016/j.vetpar.2009.12.016 20056328 10.1016/j.vetpar.2009.12.016

[CR16] Marteles D, Lebrero ME, Fernández A et al (2025) Impact of single versus multiple infection on serum protein fractions in cats. Vet Res Comm 49:1–5. 10.1007/s11259-025-10724-w 10.1007/s11259-025-10724-w PMC11971170 40183830

[CR17] Menezes-Souza D, Mendes TADO, Nagem RAP, Santos TTDO et al (2014) Mapping B-cell epitopes for the peroxidoxin of *Leishmania* (*Viannia*) *Braziliensis* and its potential for the clinical diagnosis of tegumentary and visceral leishmaniasis. PLoS ONE 9:e99216. 10.1371/journal.pone.0099216 24921246 10.1371/journal.pone.0099216 PMC4055673

[CR18] Miró G, Troyano A, Montoya A, Fariñas F et al (2018) First report of *Leishmania infantum* infection in the endangered orangutan (P*ongo Pygmaeus Pygmaeus*) in madrid, Spain. Parasit Vectors 11:185. 10.1186/s13071-018-2772-1) 29554944 10.1186/s13071-018-2772-1 PMC5859647

[CR19] Montoya A, de Quadros LP, Mateo M, Hernández L et al (2016) *Leishmania infantum infection* in bennett’s wallabies (*macropus rufogriseus rufogriseus*) in a Spanish wildlife park. J Zoo Wildl Med 47:586–593. 10.1638/2014-0216.1 27468032 10.1638/2014-0216.1

[CR20] Moraleda-Berral P, Gálvez R, Martínez-Nevado E, Pérez de Quadros L et al (2025) First clinical cases of leishmaniosis in meerkats (*Suricata suricatta*) housed in wildlife parks in madrid, Spain. Parasit Vectors 18:31. 10.1186/s13071-024-06647-1) 39871352 10.1186/s13071-024-06647-1 PMC11773741

[CR21] Ortega-García MV, Salguero FJ, Rodríguez-Bertos A, Moreno I et al (2019) A pathological study of *Leishmania infantum* natural infection in European rabbits (*Oryctolagus cuniculus*) and Iberian hares (*Lepus granatensis*). Transbound Emerg Dis 66:2474–2481. 10.1111/tbed.13305 31339665 10.1111/tbed.13305

[CR22] Pennisi MG, Persichetti MF (2018) Feline leishmaniosis: is the Cat a small dog? Vet Parasitol 251:131–137. 10.1016/j.vetpar.2018.01.012 29426470 10.1016/j.vetpar.2018.01.012 PMC7130840

[CR23] Porrozzi R, Pereira MS, Teva A, Volpini AC et al (2006) *Leishmania infantum*-induced primary and challenge infections in rhesus monkeys (*Macaca mulatta*): a primate model for visceral leishmaniasis. Trans R Soc Trop Med Hyg 10:926–937. 10.1016/j.trstmh.2005.11.005 10.1016/j.trstmh.2005.11.005 16455120

[CR24] Ramírez GA, Peñafiel-Verdú C, Altimira J, García-González B, Vilafranca M (2013) Naturally acquired visceral leishmaniosis in a captive bennett’s Wallaby (*Macropus rufogriseus rufogriseus*). Vet Pathol 50:188–190. 10.1177/0300985812446155 22692623 10.1177/0300985812446155

[CR25] Rovirosa-Hernández M, de Cortes-Ortíz J, García-Orduña L, Guzmán-Gómez F, López-Monteon D, Caba A, Ramos-Ligonio M A (2013) Seroprevalence of *Trypanosoma Cruzi* and *Leishmania Mexicana* in free-ranging howler monkeys in southeastern Mexico. Am J Primatol 75:161–169. 10.1002/ajp.22094 23165742 10.1002/ajp.22094

[CR26] Schönian G, Nasereddin A, Dinse N, Schweynoch C, Schallig HD, Presber W, Jaffe CL (2003) PCR diagnosis and characterization of leishmania in local and imported clinical samples. Diagn Microbiol Infect Dis 47:349–358. 10.1016/s0732-8893(03)00093-2 12967749 10.1016/s0732-8893(03)00093-2

[CR27] Souza TD, Turchetti AP, Fujiwara RT, Paixão TA, Santos RL (2014) Visceral leishmaniasis in zoo and wildlife. Vet Parasitol 200:233–241. 10.1016/j.vetpar.2013.12.025 24439771 10.1016/j.vetpar.2013.12.025

[CR28] Tinoco HP, da Costa MELT, Pessanha AT, Coelho CM, de Carvalho TF, Mol JPDS, Viana AG, Bueno LL, Fujiwara RT, Santos RL (2018) Visceral leishmaniasis in an infant Gorilla (*Gorilla Gorilla Gorilla)*: clinical signs, diagnosis, and successful treatment with single-dose liposomal amphotericin B. J Med Primatol 47:416–418. 10.1111/jmp.12354 29956831 10.1111/jmp.12354

[CR29] Villanueva-Saz S, Giner J, Marteles D, Verde M, Yzuel A, Riera C, Fisa R, Alcover M, Fernández A (2021) Leishmaniosis caused by *Leishmania infantum* in ferrets: update review. Vet Anim Sci 15:100229. 10.1016/j.vas.2021.100229 35028486 10.1016/j.vas.2021.100229 PMC8739881

