# Supplementary material for: Naturally acquired visceral leishmaniosis in a captive white-naped mangabey (Cercocebus lunulatus) in Spain
Source: Vet Res Commun. 2025 Sep 19;49(6):318. doi: 10.1007/s11259-025-10894-7 (PMC12449416; doi:10.1007/s11259-025-10894-7)
Supplement: Supplementary file 1 — Supplementary Material 1 (DOCX 1.86 MB) [file 11259_2025_10894_MOESM1_ESM.docx]

**Supplementary material**

**Naturally acquired visceral leishmaniosis in a White-naped mangabey in captivity**

Sergio Villanueva-Saz^1,2,3*^, María Eugenia Lebrero^1^, Jacobo Giner^1,3^, Salvador Marín Lillo^4,5^, Rafael Guerra^6^, Xavier Roca-Geronès^7^, Roser Fisa^7^, Alicia De Diego^8^, Pablo Quilez^1,2,3^, Álex Gómez^1,2*^, Diana Marteles^1,2,3^

^1^ Departamento de Patología Animal, Universidad de Zaragoza, Zaragoza, Spain

^2^ Instituto Agroalimentario de Aragón-IA2 (Universidad de Zaragoza-CITA), Zaragoza, Spain

^3^ Clinical Immunology Laboratory, Universidad de Zaragoza, Zaragoza, Spain

^4^ AAP Primadomus, Alicante, Spain

^5^ Wales Ape & Monkey Sanctuary, Caehopkin, Abercrave, United Kingdom

^6^ Centro De Conservación Zoo Córdoba, Córdoba, Spain

^7^ Departamento de Biología, Sanidad y Medioambiente, Universitat de Barcelona, Barcelona, Spain

^8^ Centro de Investigación Biomédica de Aragón (CIBA), Instituto Aragonés de Ciencias de La Salud (IACS), Zaragoza, Spain

*Corresponding author: Álex Gómez (AG) and Dr. Sergio Villanueva-Saz (SVS). Departamento de Patología Animal, Universidad de Zaragoza, C. de Miguel Servet, 177, 50013 Zaragoza, Spain. Telephone: (+34) 876554288. E-mail: [a.gomez@unizar.es](mailto:a.gomez@unizar.es) (AG); svs@unizar.es (SVS).


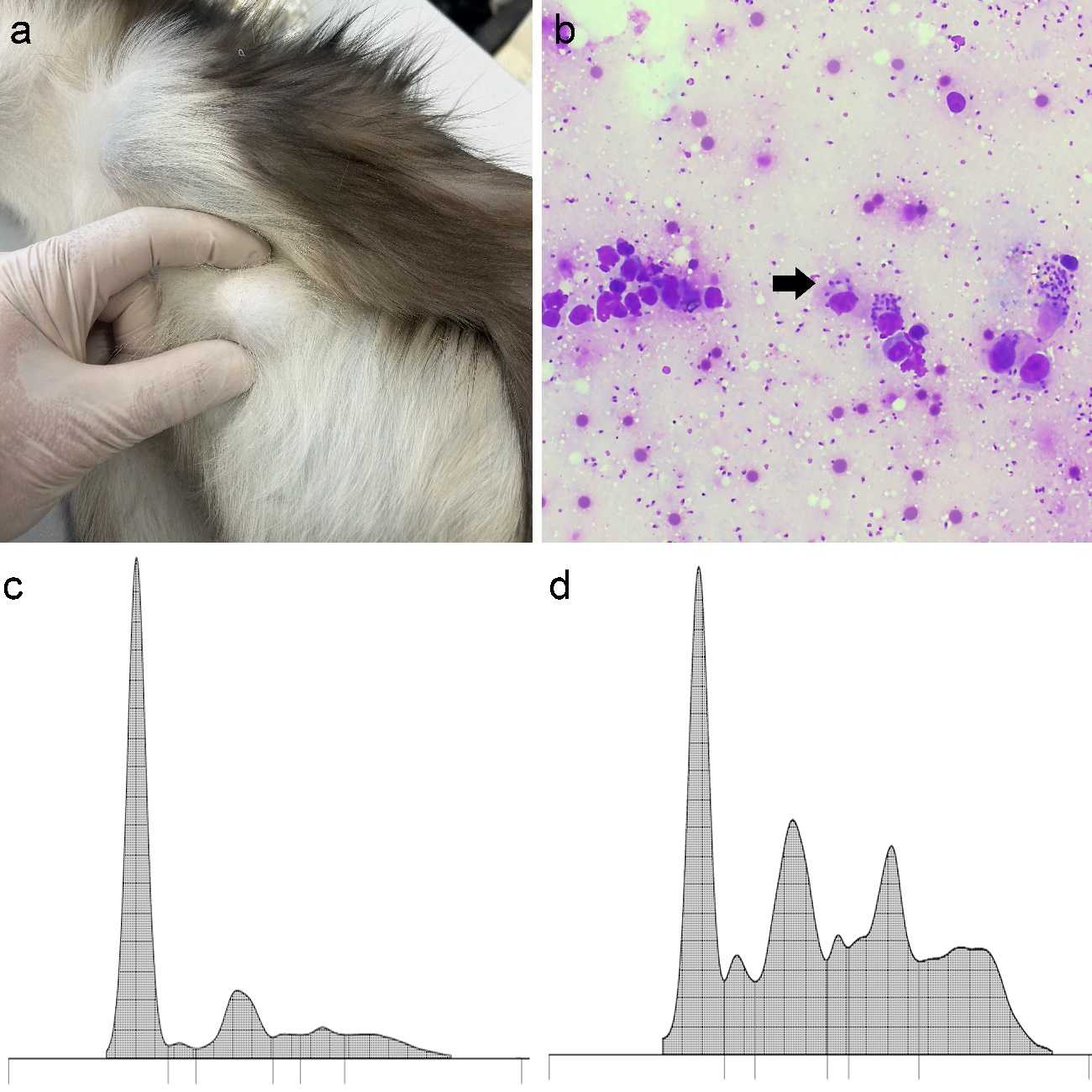


**Supplementary material S1**. Clinical findings in a captive White-naped mangabey with visceral leishmaniosis caused by *Leishmania infantum*. a) Axillary lymphadenomegaly. b) *Leishmania spp.* amastigotes within the cytoplasm of macrophages (arrow). Diff-Quick stain. c) Normal electrophoretic patter detected in a healthy White-naped mangabey. d) Acute-phase response and polyclonal gammopathy in affected White-naped mangabey.


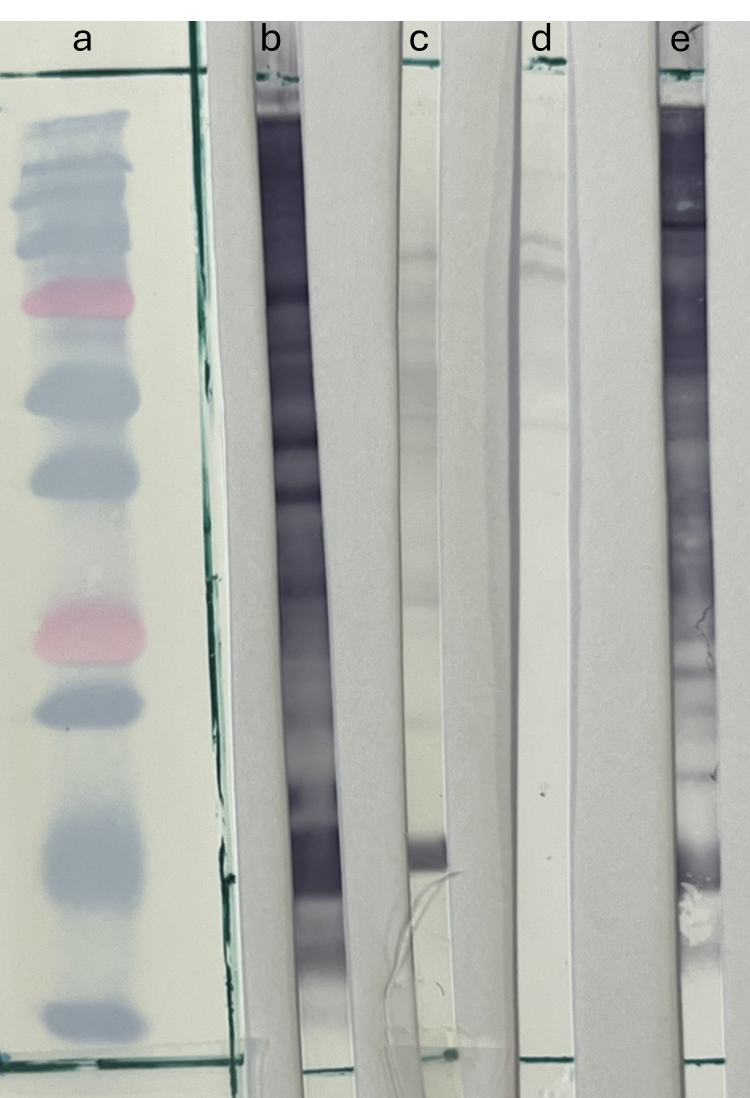


**Supplementary material S2**. Western blot technique. a) Standard. b) High positive control. c) Low positive control. d) Healthy White-naped mangabey showing no immunoreactivity against *L. infantum*. e) White-naped mangabey exhibiting immunoreactivity against *L. infantum* antigen fractions of 14, 16, 18, 20, 24, 28, 30, 34, 36, 44, 46, 48, 50, 53, 55, 58, and 71 kDa.
